# Editorial: Physical and medical conditions associated with autism

**DOI:** 10.3389/fpsyt.2024.1447188

**Published:** 2024-06-21

**Authors:** Martina Micai, David Saldaña, Mila Vulchanova, Valentina Riva

**Affiliations:** ^1^ Coordinamento e Promozione della Ricerca, Istituto Superiore di Sanità, Rome, Italy; ^2^ Laboratorio de Diversidad Cognición y Lenguaje, Departamento de Psicología Evolutiva y de la Educación, Universidad de Sevilla, Seville, Spain; ^3^ Language Acquisition and Language Processing Lab, Department of Language and Literature, Norwegian University of Science and Technology (NTNU), Trondheim, Norway; ^4^ Scientific Institute, IRCCS E. Medea, Child Psychopathology Unit, Lecco, Bosisio Parini, Italy

**Keywords:** autism, physical health, mental health, comorbidity, dual diagnosis

This Research Topic, entitled “Physical and Medical Conditions Associated with Autism,” aims to provide evidence and data to enhance our understanding of the prevalence, causes, interventions, and long-term impacts of physical and medical conditions in autistic individuals. The aim is to address these and other related questions, while raising awareness of how associated physical and medical conditions affect the lives of autistic individuals.

Certain physical and medical conditions, such as sleep-wake disorders, epilepsy, and sensory impairments, are well-recognized as being more common among autistic individuals compared to the general population. However, the prevalence and impact of other conditions, including cardiovascular issues, immune dysregulation and inflammation, genetic conditions, and constipation remain to be thoroughly established. The presence of these co-occurring conditions can hinder the success of treatments and worsen the quality of life for both autistic individuals and their careers. However, there are substantial gaps in the literature regarding these co-occurring conditions in autistic individuals.

The Research Topic is now published and includes nine different papers written by colleagues/samples from Europe (Spain, Italy, Netherlands, Romania), Asia (China and Taiwan), America, and Australia. This Research Topic consists of five original articles, three review articles, and one brief research report, published in 2022, 2023, and 2024.

## 1 Genetic and molecular mechanisms

This study by Zhou and Gao (2022) investigates the cuproptosis signaling pathway, a novel form of regulated cell death, in the context of ASD. The researchers collected gene expression profiles from brain samples of ASD model mice and blood samples from humans with ASD. They identified crucial genes in the cuproptosis pathway, such as FDX1, DLAT, LIAS, and ATP7B, using machine-learning models. The study found that the artificial neural network (ANN) model had the highest accuracy, sensitivity, and specificity, suggesting its potential for early ASD identification.

## 2 Immune dysregulation and inflammation

The reviews by Erbescu et al. (2022) and Jyonouchi (2024) explore the role of immune system dysregulation in autism spectrum disorder (ASD) and the potential of anti-inflammatory therapies. Erbescu et al. (2022) highlight the complex interaction between the central nervous system and the immune system, emphasizing the disruption of cytokine levels that contribute to neuroinflammation in ASD. They discuss various immune molecules involved in antigen presentation and inflammatory responses, maternal immune activation, brain-reactive antibodies, and autoimmunity as prenatal and postnatal factors in ASD pathophysiology. Oxidative stress, mitochondrial dysfunction, and gastrointestinal inflammation are also analyzed as contributing factors. The importance of genetic and epigenetic factors linked to immune dysregulation is highlighted, suggesting their potential as biomarkers for ASD. Jyonouchi (2024) examines the potential of anti-inflammatory therapies for ASD, noting that many ASD subjects do not respond to first-line behavioral and pharmacological interventions. The analysis focuses on the role of neuroinflammation in ASD pathogenesis, influenced by genetic, epigenetic, and environmental factors. Promising results from anti-inflammatory therapies targeting metabolic changes and oxidative stress are discussed. The study highlights the potential of repurposing existing anti-inflammatory medications and the need for a deep understanding of emerging agents, such as biologic and gatekeeper blockers, tailored to specific inflammatory pathways in ASD patients.

## 3 Epidemiological and health profiles

The study by Vidriales-Fernández et al. (2023) provides an epidemiological analysis of the health 2,629 registries of autistic individuals in Spain. It highlights a higher prevalence of nervous system disorders, mental health diagnoses, and other co-occurring conditions among autistic individuals. The study identifies increased health risks for women, older individuals, and those with intellectual disabilities, noting a high use of psychopharmacological treatments from early childhood.

Lee‘s study examines the link between early childhood constipation and the risk of developing ASD, using a nationwide population-based cohort in Taiwan. The research found that constipated 3 year-old children had a significantly higher incidence of ASD compared to non-constipated children. The study highlights the potential role of gut microbiota alterations in ASD pathogenesis and emphasizes the importance of monitoring gastrointestinal health in early childhood.

Distefano’s et al. (2023) study investigates the prevalence and impact of sleep disorders in 163 preschool autistic children. Children with poor sleep had higher scores in all areas assessed by the Children’s Sleep Habits Questionnaire (CSHQ) and on the Child Behavior Checklist (CBCL) across all domains. The analysis showed that severe sleep disorders were linked to higher scores in internalizing, externalizing, and total problems on the CBCL syndromic scales, as well as on all DSM-oriented CBCL subscales. Additionally, the study found that the connection between sleep disorders and restricted and repetitive behaviors (RRBs) is explained by anxiety-related symptoms. The findings highlight the need for screening and early intervention for sleep problems.

## 4 Treatment and strategies

Liu’s et al. (2023) systematic review and meta-analysis evaluates the efficacy of non-invasive brain stimulation (NIBS) in ASD. The analysis of 22 randomized controlled trials shows positive effects of NIBS on repetitive behaviors, cognitive function, and executive function in autistic individuals. The findings call for more rigorous, large-scale studies to validate its efficacy and establish standardized treatment protocols.

Warreman et al. (2024)’s study explores the prevalence of metabolic syndrome (MetS) in 17,705 adults with autistic traits. The research finds that MetS is more common in females with high autistic traits compared to those with low traits, while no significant difference is observed in males. The study identifies associations between MetS and poorer self-reported health, reduced physical activity, and altered leukocyte counts. The findings underscore the importance of cardiovascular prevention strategies tailored to individuals with autistic traits, particularly females.

Yau et al. (2024)’s research investigates the barriers to effective use of augmentative alternative communication (AAC) devices for minimally verbal or nonspeaking Australian autistic individuals. Through semi-structured interviews and focus groups with 30 parents, educators, and clinicians, the study identifies common themes such as stakeholder knowledge, attitudes, stigma, resource availability, AAC-user engagement, and device fit. The research highlights contrasting perspectives among stakeholders, particularly regarding stigma, resource struggles, and communication processes.

## 5 Conclusions

In summary, increased awareness of specific physical and medical conditions associated with autism enables targeted interventions and better outcomes. Comprehensive medical assessments of autistic individuals are crucial to clarify their co-occurring conditions, identify differential diagnoses, and decide on tailored, individualized interventions.

## Author contributions

MM: Conceptualization, Data curation, Writing – original draft, Writing – review & editing. DS: Writing – review & editing. MV: Writing – review & editing. VR: Writing – review & editing.

